# Immediate impact of rapid maxillary expansion on upper airway dimensions and on the quality of life of mouth breathers

**DOI:** 10.1590/2176-9451.20.3.043-049.oar

**Published:** 2015

**Authors:** Edna Namiko Izuka, Murilo Fernando Neuppmann Feres, Shirley Shizue Nagata Pignatari

**Affiliations:** 1 Postgradute student of Otolaryngology, Universidade Federal de São Paulo (UNIFESP), São Paulo, São Paulo, Brazil; 2 Assistant professor of Orthodontics, Universidade São Francisco (USF), Bragança Paulista, São Paulo, Brazil; 3 Professor of Otolaryngology, Universidade Federal de São Paulo (UNIFESP), São Paulo, São Paulo, Brazil

**Keywords:** Mouth breathing, Palatal expansion technique, Cone-beam computed tomography, Quality of life questionnaire

## Abstract

**OBJECTIVE:**

To assess short-term tomographic changes in the upper airway dimensions and quality of life of mouth breathers after rapid maxillary expansion (RME).

**METHODS:**

A total of 25 mouth breathers with maxillary atresia and a mean age of 10.5 years old were assessed by means of cone-beam computed tomography (CBCT) and a standardized quality of life questionnaire answered by patients' parents/legal guardians before and immediately after rapid maxillary expansion.

**RESULTS:**

Rapid maxillary expansion resulted in similar and significant expansion in the width of anterior (2.8 mm, p < 0.001) and posterior nasal floor (2.8 mm, p < 0.001). Although nasopharynx and nasal cavities airway volumes significantly increased (+1646.1 mm^3^, p < 0.001), oropharynx volume increase was not statistically significant (+1450.6 mm^3^, p = 0.066). The results of the quality of life questionnaire indicated that soon after rapid maxillary expansion, patients' respiratory symptoms significantly decreased in relation to their initial respiratory conditions.

**CONCLUSIONS:**

It is suggested that RME produces significant dimensional increase in the nasal cavity and nasopharynx. Additionally, it also positively impacts the quality of life of mouth-breathing patients with maxillary atresia.

## Abstract (pt)

**OBJETIVO:**

avaliar, por meio de tomografias, as mudanças em curto prazo nas vias aéreas superiores e na qualidade de vida em pacientes respiradores bucais, após expansão rápida da maxila (ERM).

**MÉTODOS:**

foram avaliados 25 pacientes respiradores bucais com atresia maxilar, com idade média de 10,5 anos, por meio de tomografia computadorizada de feixe cônico (TCFC) e questionário padronizado de qualidade de vida submetido aos pais/responsáveis, antes e imediatamente após a ERM.

**RESULTADOS:**

a ERM promoveu uma expansão, de forma semelhante e significativa, tanto na largura do soalho nasal anterior (2,8mm, p < 0,001) quanto na largura do soalho nasal posterior (2,8mm, p < 0,001). No volume aéreo da nasofaringe e fossas nasais, houve aumento significativo (+1646,1mm^3^ p < 0,001); entretanto, no volume aéreo da orofaringe, houve aumento não significativo (+1450,6 mm^3^ p = 0,066). Os resultados do questionário de qualidade de vida indicaram melhora significativa na qualidade de vida dos pacientes após a ERM, em comparação ao questionário inicial.

**CONCLUSÕES:**

a ERM promoveu aumento dimensional significativo nas fossas nasais e na nasofaringe, bem como melhorou significativamente a qualidade de vida dos pacientes.

## INTRODUCTION

Chronic mouth breathing appears to play an important role in the development of craniofacial structures, leading to significant malocclusions and craniofacial abnormalities such as increased facial height, high palate vault, maxillary atresia, and posterior crossbite.^1^^,^ 2 Transverse maxillary arch deficiency might be considered one of the most frequent occlusal alterations observed in these individuals.^3^ If pronounced, maxillary constriction might lead to posterior crossbite which hardly spontaneously reverts.^4^ Thus, whenever crossbite is observed, early orthodontic intervention is recommended.^5^

One of the procedures mostly indicated for correction of posterior crossbite is rapid maxillary expansion (RME).^6^^,^ 7 ^,^ 8 Although forces arising from RME are primarily directed to result in the opening of the midpalatal suture, adjacent facial sutures are also affected.^9^ Hence, the transverse benefit of RME might be observed not only for maxillary arch dimensions,^8^ but also for the nasal cavity, as observed by various authors.^7^^,^ 10 ^-^ 14 Most of these studies have frequently demonstrated significant increase in the cross-sectional dimensions of the nasal cavity,^7^^,^ 13 ^,^ 15 ^,^ 16 as well as volumetric increase^12^^,^ 14 ^,^ 17 and reduction in nasal resistance.^10^^,^ 14

Despite abundant evidence on nasal structure increase and reduction in resistance after RME, only a few attempts have been made to investigate whether such changes are capable of causing significant improvements on respiration, physical activities and quality of life of mouth breathers.^10^^,^ 14

One of the exams currently used^13^^,^ 15 ^,^ 16 to investigate volumetric changes after RME is cone-beam computed tomography (CBCT), which enables tridimensional assessment with satisfactory reliability, precision and accuracy,^12^^,^ 16 ^,^ 17 with low-level doses of radiation.

The aim of this study was to assess, by means of cone-beam computed tomography (CBCT), the short-term effects produced by RME on upper airway dimensions, as well as to investigate the impact of this therapy on the quality of life of mouth breathers with maxillary atresia by means of a questionnaire.

## MATERIAL AND METHODS

This non-controlled clinical trial was previously approved by Universidade Federal de São Paulo Institutional Review Board (protocol #1412/10). The study sample comprised 25 mouth breathers, males and females, aged between 6 and 13 years old and consecutively selected from the Pediatric Otorhinolaryngology Clinic of the institution where this research was carried out. In selecting the sample, the following inclusion criteria were applied: children should have presented maxillary atresia and posterior crossbite, as revealed by clinical examination performed by a single experienced orthodontist.

In order to check for the mouth breathing pattern, all patients were clinically examined by a single experienced otorhinolaryngologist. The presence of nasal obstruction was verified after anterior rhinoscopy, oroscopy and nasofiberendoscopy.

Children who had been previously subjected to orthodontic treatment were not considered as part of the study. Individuals with insufficient eruption of first permanent molar, which would prevent proper fitting of orthodontic bands, potential candidates for adenoidectomy or adenotonsillectomy, and patients with craniofacial syndromes or severe dysplasia were also dismissed. All patient's parents/legal guardians, for those who agreed to take part in the study, signed an informed consent form after proper explanation of the objectives, procedures, risks, discomforts and benefits of the research.

Firstly, parents or legal guardians were requested to answer a standardized questionnaire originally designed to measure the impact of adenotonsillectomy on the quality of life of patients with sleep breathing disorders.^18^^,^ 19 This questionnaire was conducted by a single researcher and comprised six domains concerning physical suffering, sleep disturbance, speech or swallowing problems, emotional distress, activity limitation, and degree of parents/legal guardians' concern about their own child's snoring. The scale for each answer ranged from zero to five, and referred to the frequency each symptom was perceived by parents and/or legal guardians (0= never; 1= hardly ever; 2= sometimes; 3= often; 4= very much; 5= always). All scores were summed up and total score was also analyzed.^18^^,^ 19

Afterwards, patients were referred to their first CBCT scan, (Kavo i-Cat^(r)^, settings: 8 mA, 120 kVp, 0.3 mm voxel resolution for 20 seconds). During tomographic recording, patients remained still, in sitting position, with Frankfort horizontal plane of orientation parallel to the ground.

Subsequently, children were subjected to RME with modified Biederman type appliance. At the appliance installation session, four activations (1/4 turn for each activation, 0.25 mm) were performed, and other two daily activations were made until overcorrection (when the palatal cusps of maxillary posterior teeth touches the buccal cusps of lower posterior teeth).

Immediately after overcorrection, children underwent a second tomographic examination performed under the same aforementioned conditions. Parents/legal guardians answered the same quality-of-life questionnaire, conducted by the same interviewer.

Both CBCT examination files (before and after treatment) were converted into DICOM (Digital Imaging Communication in Medicine) format, and Dolphin^(r)^ 3D software was used to read and evaluate patients' upper airways.

**Figure 1.**
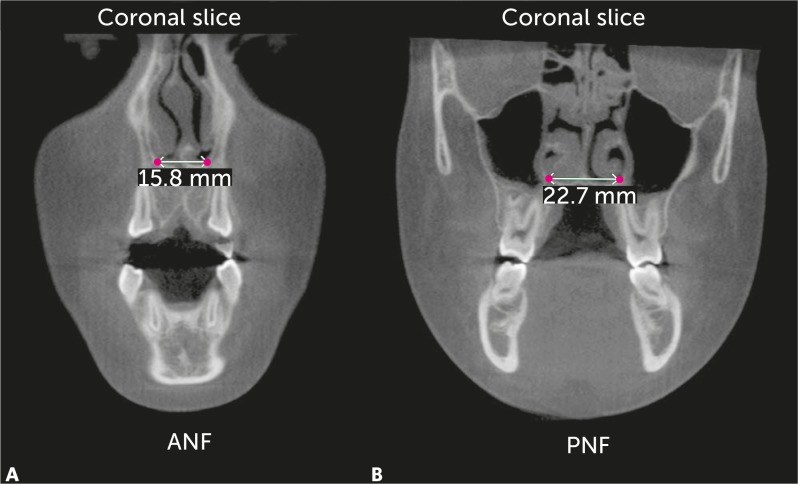
Measurement of A) anterior portion of the nasal floor (ANF) and B) posterior portion of the nasal floor (PNF).

The transverse width of the anterior portion of the nasal floor (ANF) was assessed after demarcating two points on the left and right edges of the nasal floor in the region of canines.^20^ Analysis of the transverse width of the posterior portion of the nasal floor (PNF) was performed after demarcating two points on the right and left edges of the nasal floor in the region of first permanent molars (Fig 1).^13^^,^ 20

**Figure 2.**
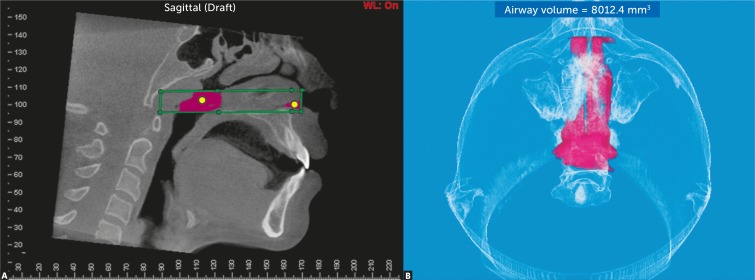
Measurement of VNN: A) limits of the nasopharynx and nasal cavities (green), and B) airway volume calculation (pink).

In order to measure the airway volume of the nasopharynx and nasal cavities (VNN), the following anatomic limits were demarcated: the upper limit was defined as the last axial slice before the fusion of the nasal septum with the pharyngeal wall observed at sagittal view; the lower limit was defined as the palatal plane, that is, a line connecting anterior and posterior nasal spines, extending to the posterior pharyngeal wall; the posterior limit was defined as the posterior pharyngeal wall; and the anterior limit was defined as the nasal cavities^21 ^(Fig 2).

**Figure 3.**
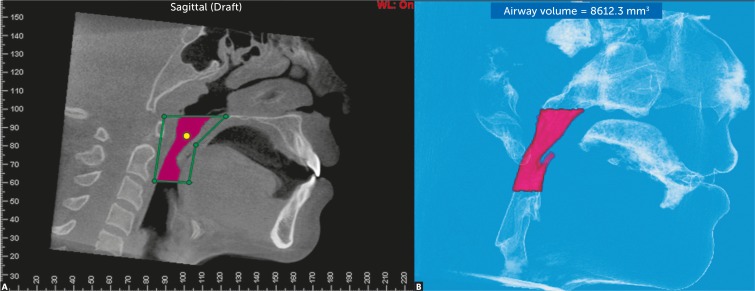
Measurement of VO: A): Limits of the oropharynx (green), and B): Airway volume calculation (pink).

In order to assess the airway volume of the oropharynx (VO), the palatal plane, extending to the posterior pharyngeal wall, represented the upper limit.^22^ The lower limit of the oropharynx was determined by a line parallel to the palatal plane, passing through the most anterior point of the second cervical vertebra^21^ (Fig 3).

Variables were considered reliable, according to the intraclass correlation coefficient (ICC) calculated after repeated readings (ANF: ICC = 0.95, p < 0.001; PNF: ICC = 0.94, p < 0.001; VNN: ICC = 0.87, p < 0.001; VO: ICC = 0.84, p < 0.001).

## Statistical analysis

Descriptive analysis of demographic data and all variables was performed. Inferential analyses were performed according to Student's t-test for paired samples and compared ANF, PNF, VNN and VO before and after RME. Wilcoxon test compared the scores for each section and the total score of the questionnaire reported before and after RME.

Statistical analysis was performed with the program "R" (version 2.15.2). In all findings obtained by inferential analyses, significance level was set at α = 5 %.

## RESULTS

The sample comprised 25 patients, 14 females (56%) and 11 males (44%),with a mean age of 10.5 years old (7.1 - 14.3; standard deviation: 2.2). Of all patients, 72% (18/25) had undergone adenoidectomy or adenotonsillectomy before the study period. Despite this fact, most patients (16/18) still had symptoms or complaints of chronic nasal obstruction. The remaining patients (7/25) were under clinical treatment for other causes of nasal obstruction.

There was statistically significant increase in ANF, PNF and VNN after treatment (Table 1). The magnitude of VO, however, showed no statistically significant difference when compared to the volume observed before RME.

**Table 1.** Comparison of ANF, PNF, VNN and VO before and after RME.

Variables	Time	Mean	Standard deviation	Mean difference	Student’s t-test (p value)
ANF (mm)	Before	16.3	1.7	+ 2.8	< 0.001
	After	19.1	1.8		
PNF (mm)	Before	22.6	2.5	+ 2.8	< 0.001
	After	25.4	3.0		
VNN (mm^3^)	Before	6114.4	3490.4	+ 1646.1	< 0.001
	After	7760.5	3841.4		
VO (mm^3^)	Before	6378.2	2357.5	+ 1450.6	0.066
	After	7828.8	4109.9		

The questionnaire total score obtained after RME was statistically lower than that obtained before RME (Table 2). Detailed analysis of results suggests that the severity of the following respiratory symptoms reduced after RME: nasal obstruction, daytime tiredness, lack of breath, snoring, choking or smothering, restless sleep, difficulty in waking up, sinking of the chest, poor pronunciation, inattention, ridiculed by snoring and school performance (Table 2). Thus, most items (physical suffering, sleep disturbance, speech and swallowing problems, emotional distress and parental concern about snoring) showed significant changes after RME (Table 2).

**Table 2.** Comparison between scores and sub-items before and after RME (Wilcoxon test P values).

Domain	Sub-items				
Physical suffering <0.001	Nasal obstruction <0.001	Daytime tiredness 0.002	Failure to gain in weight 0.079	Poor breath <0.001	-
Sleep disturbance <0.001	Snoring <0.001	Choking/ gasping for air 0.038	Restless sleep 0.001	Difficulty awakening 0.021	Chest caving in with breathing 0.042
Speech or swallowing problems 0.048	Difficulty in swallowing solid food 0.500	Choking 0.019	Muffled speech 0.248	Nasal sounding speech 0.207	Poor pronunciation 0.032
Emotional distress	Irritability	Impatience	Poor appetite	Cannot pay attention	Made fun of because of snoring
0.006	0.104	0.053	0.079	0.035	0.019
Activity limitations	Playing	Participating in sports	Doing things with friends/ family	Attending school	School performance
0.114	0.090	0.500	0.159	0.500	0.048
Parent’s concern about snoring					
0.001					
					Total Score
					< 0.001

## DISCUSSION

RME was first described by Angell^23^ in 1860 and it is a well-established and widely accepted procedure.

Of all studies available, many have emphasized the ability of RME to produce lateral expansion of the nasal cavity and to decrease nasal resistance.^8^^,^ 10 ^,^ 14 ^,^ 24 In the present research, CBCT analysis confirmed significant cross-sectional increase in both anterior and posterior regions of the nasal floor. This finding confirms the results widely observed in both postero-anterior X-ray^25^ and tomographic scans.^13^ ^,15.16,^ 17 ^,^ 20 In comparison to the dimensional increase reported herein (2.8 mm on average), studies investigating similar parameters^13^^,^ 15 ^,^ 16 ^,^ 20 reported lower (1.8 mm - 2.78 mm) transverse expansion of the nasal floor, but with no clinically significant difference.

According to the literature, the anatomical enlargement of the nasal cavity might be considered the reason for the decrease in nasal airway resistance, a commonly reported finding.^10^^,^ 14 However, there have been few attempts to assess whether any dimensional changes would lead to significant impact on the subjective impressions of patients undergoing RME.^10^^,^ 14 The data collected in our study suggest a significant respiratory improvement referred by a considerable part of the patients undergoing RME. Moreover, this research demonstrated significantly positive impact on patients' quality of life in regard to various aspects related to the obstructive respiratory condition. The increase in airway volume of the nasopharynx and nasal cavities may have contributed to the reporting of this improvement.

Studies on lateral radiographs^25^ or conventional tomography^26^ have already demonstrated significant increases in area and volume of the nasopharynx, which is consistent with the data of this research. However, Pangrazio-Kulberch et al^20^ did not achieve similar results. This difference may be related to sample discrepancies, since subjects in that research^20^ were older than the children in this study (12.6 - 13.5 *versus* 10.5). When performed in younger patients, RME is able to produce greater and more stable nasal transverse skeletal changes.^7^^,^ 11 No studies comparing skeletal changes in the nasopharynx of different age groups were found. However, evidence of greater nasal expansions in younger patients^7^^,^ 11 suggests increased likelihood of RME to produce significant skeletal results in the nasopharynx of younger individuals.

Oropharyngeal airway constrictions have been responsible for playing a significant role in the pathophysiology of obstructive sleep apnea^27^ due to association with low tongue posture, a common feature in patients with maxillary atresia.^28^ In the present research, as demonstrated by others,^16^^,^ 29 ^,^ 30 no significant increases in the airway volume of the posterior oropharynx were noted. Lack of RME impact on the size of the oropharynx was expected not only because of previous research results,^16^^,^ 29 ^,^ 30 but also due to the remote anatomical relationship between the oropharynx and the maxillary complex. Data collected herein confirm inferences that the effect of RME in the upper airway is mainly local and decreases as it "descends" in the upper airway,^30^probably due to adaptation of soft tissues.

One limitation of this study is lack of a control group. Further, the short-term follow-up (about three weeks) limited the authors' ability to infer potential permanent benefits that RME could bring to the dimensions of the airway. Langer et al^31^ observed that the decrease in nasal resistance observed shortly after RME did not persist throughout 30 months. Still, one cannot underestimate the clinical value of short-term effects of RME on the immediate relief of respiratory symptoms, which was substantially demonstrated in this research. It is the authors' opinion that RME, even if transiently affecting the upper airway, should be regarded as an essential therapeutic approach for patients with upper airway disturbances. Because of the substantial respiratory improvements reported by patients in this study, it is suggested that controlled clinical trials be conducted in order to assess subsequent respiratory effects of RME in patients with obstructive complaints.

## CONCLUSION

Short-term RME promotes significant increase in airway volume of the nasopharynx and nasal cavities as well in anterior and posterior widths of the nasal floor. Additionally, it significantly improves the quality of life of mouth-breathing patients with maxillary atresia.
